# Glycemic control and diabetes management in hospitalized patients in Brazil

**DOI:** 10.1186/1758-5996-5-62

**Published:** 2013-10-18

**Authors:** Edson Duarte Moreira Jr, Patricia Carvalho Balthazar Silveira, Raimundo Celestino Silva Neves, Clodoaldo Souza Jr, Zaira Onofre Nunes, Maria da Conceição C Almeida

**Affiliations:** 1 Clinical Research Center, Charitable Works Foundation of Sister Dulce, Av. Bonfim 161, Salvador, Bahia, Brasil 40.415-000; 2 Division of Cancer Epidemiology, McGill University, 546, Pine Avenue West, Montreal, Quebec, Canada H2W 1S6; 3 Gonçalo Moniz Research Center, Oswaldo Cruz Foundation, Brazilian Ministry of Health, Rua Waldemar Falcão 121, Salvador, Bahia, Brasil 40.296-710

**Keywords:** Glycemic control, Inpatients, Hospitals, Diabetes mellitus, Epidemiology, Brazil

## Abstract

**Background:**

The importance of tight blood glucose control among outpatients with diabetes mellitus is well established, however, the management of diabetes in the hospital setting is generally considered secondary in importance. This study sought to assess glycemic control and diabetes management in adult patients admitted to hospitals in Brazil.

**Methods:**

A cross-sectional and nationwide survey was conducted from July 2010 to January 2012. Eligible cases were 18 years of age or older, had a diagnosis of diabetes and a hospitalization length of stay ≥72 hours. Socio-demographic information, hospitalization details, and data on diabetes diagnosis, management and treatment were collected for all patients by chart review. Information on all blood glucose (BG) readings for a maximum of 20 consecutive days of hospitalization was recorded for each patient.

**Results:**

Overall, 2,399 patients were surveyed in 24 hospitals located in 13 cities from all five Brazilian regions. The prevalence of patients presenting hyperglycemic (BG >180 mg/dL) or hypoglycemic (BG <70 mg/dL) events was 89.4% and 30.9% in patients in general wards, and 88.2% and 27.7% in those in Intensive Care Units (ICUs), respectively. In addition, a BG measure >180 mg/dL was recorded in two-thirds of the patient-days. A high proportion of patients were treated with sliding-scale insulin regimen alone in the general wards (52.0%) and in the ICUs (69.2%), and only 35.7% and 3.9% received appropriate insulin therapy in general wards (basal + bolus insulin) and in ICUs (continuous IV insulin), respectively.

**Conclusions:**

Inpatient glycemic control and diabetes management needs improvement. Opportunities to improve care in Brazilian hospitals include expanded use of intravenous insulin and subcutaneous basal-bolus insulin protocols, avoiding use of sliding-scale insulin alone, increased frequency of blood glucose monitoring, and institution wide quality improvement efforts targeting both physician and nursing behavior.

## Background

The importance of tight blood glucose control among outpatients with type 1 and 2 diabetes mellitus is well established [1,2]. Over the past years, there has been increased attention to inpatient glycemic control, as evidenced by numerous reports published in the medical community [3-5]. Compelling evidence continues to accumulate suggesting that poorly controlled blood glucose levels among inpatients are associated with increased morbidity and mortality, as well as with higher health care costs [6-9]. In the past decade, studies have focused attention to the possibility that hyperglycemia in the hospital is not necessarily a benign condition and that aggressive treatment of diabetes and hyperglycemia may result in reduced mortality and morbidity [10-12]. In fact, hyperglycemia is a strong predictor of adverse clinical outcome in a range of diseases such as acute stroke [13,14], congestive heart failure [15,16], community acquired pneumonia [17], acute myocardial infarction [18,19] and postoperative nosocomial infection [20].

Based upon these findings, the American College of Endocrinology (ACE) and the American Diabetes Association (ADA) have published guidelines recommending tight glucose control for inpatients with diabetes [21,22]. They also recommended the use of continuous insulin infusion given through a standardized protocol as the approach to control hyperglycemia in critically ill inpatients. For noncritical ill diabetic inpatients, they suggested the use of specific insulin regimens with combined basal and short-acting insulin and appropriate bedside glucose monitoring, avoiding the use of sliding-scale insulin (fast or rapid-acting insulin in response to hyperglycemia) alone. More recently, studies showing that intensive glucose control for critically ill patients is associated with severe hypoglycemia and/or increased mortality [4,5,23] have led to less stringent recommendations [7,24-26]. Research assessing inpatient glycemic control state after the development of these guidelines has shown that control is still poor and needs improvement [3,27,28].

The Brazilian Diabetes Society has endorsed the AACE/ADA recommendations on inpatient glucose control [29], but information about the epidemiology of diabetes and glycemic control in Brazil is scarce. According to a study performed by the Brazilian Ministry of Health in 1992 [30], diabetes was the fifth most common reason for hospitalizations and ranked among the ten major causes of mortality. Moreover, in a large multicenter survey in Brazil, the prevalence of inadequate glycemic control of outpatients with type 1 or type 2 diabetes was 90% and 73%, respectively [31]. Thus, outpatient diabetes management is a major problem in Brazil, with an impact on public heath comparable to that in other countries worldwide [32-35]. Knowledge on current state of inpatient glycemic control in Brazil is essential for planning healthcare programs targeting improved diabetes control. The purpose of this study was to describe glycemic control and diabetes management in hospitalized patients in Brazil.

## Methods

This cross-sectional and nationwide survey was conducted in Brazil from July 2010 to January 2012. It was designed to portray glycemic control and diabetes management in a sample of adult patients admitted to hospitals in Brazilian urban areas. Study design and reporting format are in accordance with the recommended STROBE (Strengthening the Reporting of Observational Studies in Epidemiology) guidelines [36].

### Site selection

Patients were surveyed in hospitals located in 13 cities belonging to all five Brazilian regions, as follows: Southeast (Belo Horizonte, Botucatu, Marilia, Rio de Janeiro and São Paulo), South (Campina Grande do Sul, Caxias do Sul, Curitiba, Porto Alegre), Mid-west (Brasília), Northeast (Salvador and Fortaleza) and North (Belém). For the site selection, we identified in each of the participating cities a list of candidate hospitals, to be chosen from those with longer experience in clinical research and epidemiological surveys. Each participating medical center had to be a general hospital, medium to large size (>50 beds), with registry of primary and secondary diagnosis for all inpatients (preferably in an electronic database), and had to have medical chart archives accessible to study data collectors in order to gather information from patient charts. According to these criteria, 30 hospitals were invited to participate in the study. Twenty four accepted and six declined for administrative reasons. Hospitals joining the study were classified as academic (7), public (6), or private (11).

### Study population

Patients who met the eligibility criteria were consecutively included in the study in reverse chronological order proceeding back in time until the target number of patients per site was reached. Eligible patients had to be ≥18 years of age, have a known diagnosis of diabetes (type 1 or type 2) either prior to admission or during the hospitalization, and have a 72-hour or longer length of stay in the hospital. Patients who had been admitted for diabetic ketoacidosis, hyperosmolar hyperglycemic state or gestational diabetes, who had a history of pancreatic transplant, or patients on hospice or palliative care during hospital admission were not included. Each hospital was asked to enroll at least 80 and no more than 120 patients. The study protocol was approved by Ethical Review Boards in each respective city.

### Data collection

Socio-demographic information, hospitalization details, and data on diabetes diagnosis, management and treatment (type and route of insulin administration) were collected for all patients by chart review using a structured questionnaire. Information on all blood glucose (BG) readings for a maximum of 20 consecutive days of hospitalization was recorded for each patient. Measurement day 1 was defined as the day of admission for cases with previously known diabetes or as the day of diagnosis for patients diagnosed with diabetes during the hospitalization. Glucose measurements were recorded for each measurement day as available, both bedside (capillary blood glucose) and laboratory serum glucose values were utilized. Appropriate insulin therapy was defined as scheduled subcutaneous insulin that delivers basal, nutritional, and correction (supplemental) components for non-critically ill patients and as continuous intravenous insulin for critically ill patients. Glycosylated hemoglobin (A1C) values were included if they were recorded during the first week of hospitalization or within 30 days prior to admission. A team of study nurses (not part of the hospital staff) was hired and trained for the data collection by one of the investigators (EDM) at each study site. They were given orientation on the protocol and specific details concerning data abstraction, and received technical and content support during the study by the study staff. In addition, a hospital questionnaire was also completed at each site, collecting information on whether the blood glucose measures and insulin administration were recorded in the same form, existence of a protocol for the treatment of hypoglycemia, existence of a mandatory protocol of intravenous insulin infusion for intensive care unit (ICU) patients, and whether the hospital had endocrinology/diabetes team and/or ward.

### Statistical analysis

We employed two analytic approaches for reporting BG levels. One approach, the patient-day approach, grouped BG levels by calendar day for each patient, and then calculated a mean BG level for each patient-day. The other one, the patient approach, employed each patient’s mean BG level for the entire hospitalization as a single data point. For each of these approaches, some of the following performance measures were calculated: mean BG level, median BG level and the percentage of BG levels that fell within a predefined “optimal” range (80–139 mg/dL), stratified by location (general ward or ICU). Hypoglycemic (BG <70 mg/dL) and hyperglycemic (BG >180, >200 or >300 mg/dL) event rates were also determined. Statistical analyses were performed with Stata version 10 (Stata Corporation, College Station, TX).

## Results

A total of 2,399 patients was surveyed in 24 hospitals (11 private, 7 academic and 6 public) located in 13 cities from all five regions in Brazil. Our sample was comprised by a higher proportion of males than females. The age distribution was skewed towards the older categories, as nearly two-thirds of the subjects were age 60 years or older (Table 1). The Southeast and South regions contributed more patients to the study, while the North and Mid-west regions contributed fewer patients, resembling the demographic distribution of population in Brazil. Most patients had diabetes type 2 and the information on diabetes type was missing in over one third of the patients. Diabetes had been diagnosed prior to admission in nearly all patients (98.7%). In regard to preadmission diabetes medication regimen, oral antidiabetic drugs were the most common treatment reported (37.7%), followed by insulin alone (17.3%), and approximately one-quarter had no information registered on chart. The mean and median hospital lengths of stay were 14.6 and 10 days, respectively. In general, endocrine/diabetes consultation was infrequent, and patients in general wards were more likely to have such consultation than those in ICUs (Table 1).

**Table 1:** Selected characteristics (%) of 2,399 hospitalized patients with diabetes in Brazil, 2010-2012

	**Location**		**Total**
	**General ward**	**Intensive care unit**	
	**(n=1,934)**	**(n=465)**	**(n=2,399)**
***Socio demographics***			
**Age in years**			
<30	2.3	0.2	1.9
30 – 39	3.5	1.7	3.2
40 – 49	10.7	5.4	9.7
50 – 59	22.7	22.2	22.6
60 – 69	27.2	31.3	28.0
≥70	33.5	39.2	34.6
**Sex**			
Male	53.7	57.2	54.4
Female	46.3	42.8	45.6
**Region**			
Southeast	46	38	44
South	23	11	21
Northeast	19	8	17
Midwest	8	43	15
North	4	0.4	3
**Hospital type**			
Private	43	57	46
Academic	31	27	30
Public	26	16	24
***Health information***			
**Body mass index (Kg/m**^**2**^**)**			
Underweight (<18.5)	2.6	0.8	2.2
Normal weight (18.6 – 24.9)	31.5	24.7	30.0
Overweight (25.0 – 29.9)	33.5	38.7	34.6
Obese (30.0 – 39.9)	29.0	31.7	29.6
Morbidly obese (≥40.0)	3.4	4.1	3.6
**Diabetes type**			
Type 1	9.5	3.7	8.3
Type 2	55.1	62.7	56.5
Information not found in the chart	35.4	33.6	35.2
**Preadmission diabetes medication regimen**^**a**^			
None	8.9	12.8	9.6
Oral medications only	36.5	42.5	37.7
Insulin only	18.6	12.2	17.3
Insulin and oral medications	11.2	7.8	10.5
Information not found in the chart	24.8	24.7	24.9
**Mean hospital length of stay (days)**	14.9	13.2	14.6
**Median hospital length of stay (days)**	10	9	10
**Outcome**			
Discharged	94.2	85.5	92.6
Transferred	1.7	6.8	2.7
Died	4.1	7.7	4.8
**Consulted with a diabetes specialist**^**b**^	12.9	3.5	11.1

^a^Includes only patients diagnosed with diabetes prior to admission (n=2,367).

^b^Within a 3-day period after hospital admission.

Hospital performance of recommended diabetes care measures according to location (general ward or ICU) is shown in Table 2. Physician documentation of diabetes history was recorded in more than 95% of the medical charts, but 16% or less had an A1C assessment documented. Laboratory glucose assessment within 8 hours of hospital admission for diabetes patients was low in both general wards (31.2%) and ICUs (47.1%), and BG monitoring at least 4 times daily occurred in only 55.2% to 61.2% of patient-days. A relatively high proportion of patients were treated with sliding-scale insulin regimen alone in the general wards (55.2%) and in the ICUs (61.2%), only 35.7% and 3.9% received appropriate insulin therapy in general wards and in ICUs, respectively (Table 2).

**Table 2:** Diabetes management of 2,399 hospitalized patients in Brazil, 2010-2012

	**General ward**	**Intensive care unit**
	**(n=1,934)**	**(n=465)**
**Physician documentation of diabetes history in medical record**	95.4^a^	97.0
**HbA**_**1C **_**assessment documented for diabetes patients**^**b**^	16.0	11.0
**Blood glucose measured within 8 hours of admission or diabetes diagnosis**	31.2	47.1
**Blood glucose monitored at least four times daily (by patient-days)**	55.2	61.2
**Diabetes treatment**		
Sliding-scale insulin only	52.0	69.2
Short-acting and long-acting subcutaneous insulin^c^	35.7	21.1
NPH+Bolus	29.8	14.0
Basal+Bolus	5.9	7.1
Continuous IV insulin	0	3.9
Other insulin regimen	3.6	3.5
Oral agents only	4.8	1.3
Diet only	3.9	1.0

^a^Percent.

^b^Measured during first week of hospitalization or within 30 days prior to admission.

^c^Scheduled subcutaneous insulin (basal, nutritional, and correction components).

Measures used to assess the quality of glycemic control in hospitalized patients are presented in Table 3. The percentage of early morning BG readings ≤180 mg/dL on measurement day 3 was not sufficiently high for patients in general wards (54.7%) and in ICUs (62.8%). The prevalence of any hyperglycemic (BG >180 mg/dL) or hypoglycemic events (BG <70 mg/dL) was 89.4% and 30.9% in patients in general wards, and 88.2% and 27.7% in those in ICUs, respectively. In addition, a BG measure >180 mg/dL was recorded in about two-thirds of the patient-days, and approximately half of the patient-days had at least one BG value greater than 200 mg/dL. When the threshold was raised to 300 mg/dL, the percentage of any hyperglycemic events ranged from 21.1% of patient-days in general wards to 16.4% in ICUs. The percentage of patient-days with any BG value <70 mg/dL was relatively low in general wards (6.7%) and in ICUs (4.9%). Overall, the percentage of patient-days with BG values within a predefined “optimal” range (80-139 mg/dL) was only 11.8% in general wards and 15.0% in ICUs. The mean percentage of glucose readings >180 mg/dL by patient stay (entire hospitalization) was 40.2 % in general wards and 34.9% in ICUs, while the mean percentage of glucose readings <70 mg/dL by patient stay was 2.1% and 1.6%, respectively. The morning glucose mean by patient-day was 162 mg/dL in general wards and 158 mg/dL in ICUs, and the median was 141 mg/dL and 143 mg/dL, respectively (Table 3).

**Table 3:** Measures of glycemic control in 2,399 hospitalized patients in Brazil, 2010-2012.

	**General ward**	**Intensive care unit (ICU)**
Number of patients	1,934	465
% of BG on morning of D3 ≤180 mg/dL	54.7	62.8
% of patients with any BG >180 mg/dL	89.4	88.2
% of patients with any BG <70 mg/dL	30.9	27.7
Mean % glucose readings >180 mg/dL analyzed by patient stay (SD)	40.2 (27.6)	34.9 (26.1)
Mean % glucose readings <70 mg/dL analyzed by patient stay (SD)	2.1 (4.5)	1.6 (3.3)
Number of patient-days	18,887	4,227
% of patient-days with any BG >180 mg/dL	64.7	61.3
% of patient-days with any BG >200 mg/dL	55.8	52.1
% of patient-days with any BG >300 mg/dL	21.1	16.4
% of patient-days with any BG <70 mg/dL	6.7	4.9
% of patient-days with all BG within target range (80–139 mg/dL)	11.8	15.0
% of patient-days with mean BG within target range (80–139 mg/dL)	30.0	32.4
Mean of morning BG analyzed by patient-day (SD)	162 (78)	158 (66)
Median of morning BG analyzed by patient-day	141	143

BG = fingerstick blood glucose; D3= measurement day 3; SD= standard deviation.

**Figure 1:**
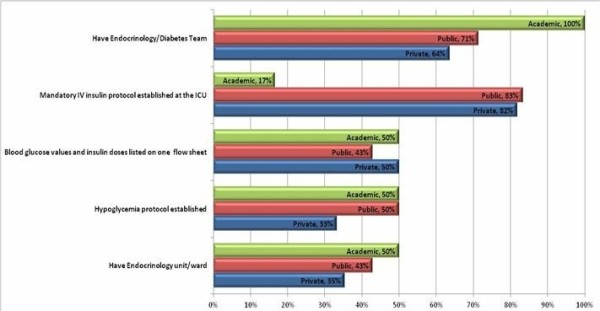
Selected characteristics of hospitals (n=24) by type: academic, public or private, Brazil, 2010-2012.

Characteristics of diabetes management in participating hospitals stratified by type: academic, public or private are shown in Figure 1. All academic hospitals had an endocrinology/diabetes team compared to 71% and 64% of public and private hospitals, respectively. A mandatory IV insulin protocol was established at the ICU in most public (83%) and private (82%) hospitals, while in only 17% of the ICU in academic hospitals. About half of the hospitals had all point-of-care blood glucose values and insulin doses for a patient listed together on one flow sheet. Similarly, roughly half of the hospitals had an established protocol to treat hypoglycemic events.

## Discussion

To our knowledge, this is the first multicenter, nationwide survey to describe glycemic control and diabetes management in hospitalized patients in Brazil. Overall, the quality of inpatient glucose management in hospitals included in our survey was poor. There were several deficiencies in the glycemic control and diabetes management of hospitalized patients in our study. These deficiencies were both in processes of care (e.g., limited use of basal and bolus insulin) and in outcomes (i.e., glycemic control) compared to current recommended guidelines [7,24]. We observed only 11.8%-15.0% of patient-days with mean BG levels between 80-139 mg/dL, though Goldberg et al. [37] have proposed 85% of patient-days with mean BG levels within this range as a “gold standard” for inpatient glycemic control.

The target of maintaining all glucose values ≤180 mg/dL recommended in the ADA/AACE guidelines for hospital diabetes management was not generally achieved [7,24]. The mean rate of hyperglycemia (BG>180 mg/dL) per patient was higher than previously reported in a survey at a large teaching hospital in the US (40.2% vs. 31%) [27]. Furthermore, up to two-thirds of patient-days had at least one BG measure above 180 mg/dL. Although hyperglycemia was common, hypoglycemia was relatively infrequent. This should be interpreted with caution, and may be a consequence of insufficient insulin regimens and loose glycemic control rather than an indication of good glycemic management, as suggested by the high rates of hyperglycemia.

Most ICUs in private or public hospitals had a mandatory IV insulin protocol established, as opposed to those in academic hospitals. Nonetheless, overall, very few patients in the ICUs were treated with continuous IV insulin. Thus, suggesting that establishment of an IV insulin protocol is not sufficient and does not guarantee its use will be necessarily mandatory. Failure to provide comprehensive training of ICU personnel about the protocol, lack of appropriate equipment or adequate glucose monitoring, and low awareness regarding the importance of glycemic control are among the potential barriers to implementing a mandatory IV insulin protocol. In addition, more than half of the hospitals in our study had not established a hypoglycemia treatment protocol yet. Poor quality of diabetes care and non-existence of glycemic management guidelines/protocols indicate that there remains potential for substantial improvements in diabetes care in hospitalized patients in Brazil. Formal communication among various professionals and services as well as appropriate training can garner support from health care providers for new practices and protocols.

Appropriate insulin therapy was given to no more than one third of inpatients with diabetes in our study. Basal insulin was prescribed for only 5.9% of patients, as compared to 43% for patients in a previous survey [27]. Moreover, most patients with diabetes in our survey received sliding-scale insulin regimen alone, even though it has been shown that sliding-scale insulin by itself is associated with poor inpatient glycemic control and even deleterious effects [38]. In a study of 999 patients with known diabetes treated in 44 hospitals across the U.S., 16% percent of patients with type 1 diabetes and 35% of patients with type 2 diabetes (using insulin as outpatients) were treated with sliding-scale insulin alone [39]. Possible barriers to adherence to the recommended standards and optimal diabetes care in our study include a fragmented delivery system, the lack of a system that facilitate the appropriate use of scheduled insulin therapy and low institution support for inpatient multidisciplinary team training.

In regard to performance of recommended hospital diabetes care practices [7,24], there was also evidence for an immediate need for improvement. Few patients had information regarding diabetes type and preadmission medication regimen recorded on their medical charts, and even less patients had an A1C measurement registered during the first week of hospitalization or within 30 days prior to admission. Thus, indicating poor quality of data recording and the need to improve practices and training of health care personnel. Two-thirds of inpatients with diabetes did not have their blood glucose measured within 8 hours of their hospital admission, and in only 55%-61% of the patient-days the BG was monitored at least four times per day. In contrast, a study conducted on 1,718 patients with a history of diabetes in thirty-seven US academic medical centers found that 31% had an A1C measurement, 77% had a laboratory blood glucose result recorded within 8 hours of hospital admission, and 81.3% had blood glucose monitored at least 4 times on the second day of hospitalization [3].

Insulin-use safety in the hospital setting has recently become a goal to quality improvement efforts [40-42]. Future research should focus on strategies to improve glucose control, as well as on patient, clinician, and system barriers to improving inpatient glycemic management, while enhancing the safe use of insulin in this setting. Factors such as competing priorities and limited resources, skepticism about the benefits of tight inpatient glycemic control, fear of hypoglycemia, inadequate knowledge and understanding of diabetes, hyperglycemia, and appropriate management of blood glucose levels may play an important role in hospital settings similar to ours.

### Strengths and limitations

Strengths of this study include its data collection methods with rigorous inclusion criteria, collection of detailed glycemic data by a team of non-staff trained personnel, and use of various statistical approaches to more accurately assess glycemic control. We also included a large sample of inpatients from private, public and academic hospitals located in all five Brazilian regions. Despite this, there are some limitations to this study. The data are retrospective and only a limited number of clinical variables could be assessed for each patient. There could also have been differences in the frequency of glucose measurement depending on treatment, which can potentially bias estimated prevalence of hyperglycemia and hypoglycemia. We also did not have a practical method to assess nutritional status or the adequacy of insulin dosing over time for each patient.

## Conclusions

Our results indicate that inpatient glycemic control and diabetes management still needs much improvement, despite the evidence of the hazards of inpatient hyperglycemia and the publication of specialist consensus guidelines on inpatient glucose management in the past decade. Opportunities to improve care in Brazilian hospitals include expanded use of intravenous insulin, subcutaneous basal insulin and scheduled nutritional insulin protocols, increased frequency of blood glucose monitoring, avoiding use of sliding-scale insulin by itself, and institution wide quality improvement efforts targeting health care personnel behavior.

## Appendix

The following is a list of co-authors, the members of the Brazilian Diabetes Investigators’ Group: Adriana C. Forti, MD; Célio C. Borges, MD; Débora V. Soares, MD; Edson P. Brum, MD; Elza M. S. Constantino, MD; Francisco A. Oliveira, MD; Freddy Eliaschewitz, MD; Hermelinda Pedrosa, MD; Jorge Gross, MD; José E. P. Oliveira, MD; Lucia Cordeiro, MD; Lúcia P. E. Souza, MD; Marcia Nery, MD; Marcos Tambascia, MD; Maria R. Calsolari, MD; Reinaldo B. M. Machado, MD; Reine Chaves, MD; Rosane Kupfer, MD; Ruy Lyra, MD; Saulo Cavalcante, MD; and Silmara Leite, MD.

## Abbreviations

BG: Blood glucose; ICU: Intensive care unit; ACE: American college of endocrinology; ADA: American diabetes association; STROBE: Strengthening the reporting of observational studies in epidemiology; A1C: Glycosylated haemoglobin; SD: Standard deviation.

## Competing interests

EDM has received grant support through his institution from Sanofi-Aventis, Merck, Pfizer and Novartis, and was compensated by Sanofi-Aventis, Merck and Pfizer for serving at Scientific Advisory Committee. All other academic co-authors have no disclosure.

## Authors’ contributions

EDM conceived the study hypothesis, supervised data analyses and wrote the manuscript, PCBS, ZON and MCCA took a lead role in the data collection. RCSN and CS contributed to the study design and to the data interpretation. PCBS made substantial contribution to data analysis and writing of the manuscript. All authors read and approved the final manuscript.

## References

[B1] The Diabetes Control and Complications Trial Research Group The effect of intensive treatment of diabetes on the development and progression of long-term complications in insulin-dependent diabetes mellitus New England J Med 1993 329 977 986 8366922 10.1056/NEJM199309303291401

[B2] UK Prospective Diabetes Study (UKPDS) Group Intensive blood-glucose control with sulphonylureas or insulin compared with conventional treatment and risk of complications in patients with type 2 diabetes (UKPDS 33) Lancet 1998 352 837 853 9742976

[B3] Boord JB Greevy RA Braithwaite SS Arnold PC Selig PM Brake H Cuny J Baldwin D Evaluation of hospital glycemic control at US academic medical centers J Hosp Med 2009 4 35 44 19140174 10.1002/jhm.390

[B4] Finfer S Chittock DR Su SY Blair D Foster D Dhingra V Bellomo R Cook D Dodek P Henderson WR Intensive versus conventional glucose control in critically ill patients New England J Med 2009 360 1283 1297 19318384 10.1056/NEJMoa0810625

[B5] Wiener RS Wiener DC Larson RJ Benefits and risks of tight glucose control in critically ill adults: a meta-analysis Jama 2008 300 933 944 18728267 10.1001/jama.300.8.933

[B6] American Diabetes A Economic costs of diabetes in the U.S. in 2007 Diabet Care 2008 31 596 615 10.2337/dc08-9017 18308683

[B7] Moghissi ES Korytkowski MT DiNardo M Einhorn D Hellman R Hirsch IB Inzucchi SE Ismail-Beigi F Kirkman MS Umpierrez GE American Association of Clinical Endocrinologists and American Diabetes Association consensus statement on inpatient glycemic control Endocr Pract 2009 15 353 369 19454396 10.4158/EP09102.RA

[B8] Umpierrez GE Isaacs SD Bazargan N You X Thaler LM Kitabchi AE Hyperglycemia: an independent marker of in-hospital mortality in patients with undiagnosed diabetes J Clin Endocrinol Metab 2002 87 978 982 11889147 10.1210/jcem.87.3.8341

[B9] Malmberg K Norhammar A Wedel H Ryden L Glycometabolic state at admission: important risk marker of mortality in conventionally treated patients with diabetes mellitus and acute myocardial infarction: long-term results from the Diabetes and Insulin-Glucose Infusion in Acute Myocardial Infarction (DIGAMI) study Circulation 1999 99 2626 2632 10338454 10.1161/01.cir.99.20.2626

[B10] van den Berghe G Wouters P Weekers F Verwaest C Bruyninckx F Schetz M Vlasselaers D Ferdinande P Lauwers P Bouillon R Intensive insulin therapy in critically ill patients New England J Med 2001 345 1359 1367 11794168 10.1056/NEJMoa011300

[B11] Lazar HL Chipkin SR Fitzgerald CA Bao Y Cabral H Apstein CS Tight glycemic control in diabetic coronary artery bypass graft patients improves perioperative outcomes and decreases recurrent ischemic events Circulation 2004 109 1497 1502 15006999 10.1161/01.CIR.0000121747.71054.79

[B12] Furnary AP Wu Y Bookin SO Effect of hyperglycemia and continuous intravenous insulin infusions on outcomes of cardiac surgical procedures: the Portland diabetic project Endocr Pract 2004 10 Suppl 2 21 33 15251637 10.4158/EP.10.S2.21

[B13] Capes SE Hunt D Malmberg K Pathak P Gerstein HC Stress hyperglycemia and prognosis of stroke in nondiabetic and diabetic patients: a systematic overview Stroke 2001 32 2426 2432 11588337 10.1161/hs1001.096194

[B14] Jorgensen H Nakayama H Raaschou HO Olsen TS Stroke in patients with diabetes. The Copenhagen stroke study Stroke 1994 25 1977 1984 8091441 10.1161/01.str.25.10.1977

[B15] Guazzi M Brambilla R De Vita S Guazzi MD Diabetes worsens pulmonary diffusion in heart failure, and insulin counteracts this effect Am J Respir Crit Care Med 2002 166 978 982 12359657 10.1164/rccm.200203-234OC

[B16] Masoudi FA Wang Y Inzucchi SE Setaro JF Havranek EP Foody JM Krumholz HM Metformin and thiazolidinedione use in medicare patients with heart failure Jama 2003 290 81 85 12837715 10.1001/jama.290.1.81

[B17] McAlister FA Majumdar SR Blitz S Rowe BH Romney J Marrie TJ The relation between hyperglycemia and outcomes in 2,471 patients admitted to the hospital with community-acquired pneumonia Diabet Care 2005 28 810 815 10.2337/diacare.28.4.810 15793178

[B18] Capes SE Hunt D Malmberg K Gerstein HC Stress hyperglycaemia and increased risk of death after myocardial infarction in patients with and without diabetes: a systematic overview Lancet 2000 355 773 778 10711923 10.1016/S0140-6736(99)08415-9

[B19] Malmberg K Ryden L Wedel H Birkeland K Bootsma A Dickstein K Efendic S Fisher M Hamsten A Herlitz J Intense metabolic control by means of insulin in patients with diabetes mellitus and acute myocardial infarction (DIGAMI 2): effects on mortality and morbidity Eur Heart J 2005 26 650 661 15728645 10.1093/eurheartj/ehi199

[B20] Grey NJ Perdrizet GA Reduction of nosocomial infections in the surgical intensive-care unit by strict glycemic control Endocr Pract 2004 10 Suppl 2 46 52 15251640 10.4158/EP.10.S2.46

[B21] Association AD Standards of medical care in diabetes Diabet Care 2005 28 s4 s36 15618112

[B22] Garber AJ Moghissi ES Bransome ED Jr Clark NG Clement S Cobin RH Furnary AP Hirsch IB Levy P Roberts R American College of Endocrinology position statement on inpatient diabetes and metabolic control Endocr Pract 2004 10 Suppl 2 4 9 15251633 10.4158/EP.10.S2.4

[B23] Finfer S Liu B Chittock DR Norton R Myburgh JA McArthur C Mitchell I Foster D Dhingra V Henderson WR Hypoglycemia and risk of death in critically ill patients New England J Med 2012 367 1108 1118 22992074 10.1056/NEJMoa1204942

[B24] Association AD Standards of medical care in diabetes–2013 Diabetes Care 2013 36 Suppl 1 S11 S66 23264422 10.2337/dc13-S011 PMC3537269

[B25] Jacobi J Bircher N Krinsley J Agus M Braithwaite SS Deutschman C Freire AX Geehan D Kohl B Nasraway SA Guidelines for the use of an insulin infusion for the management of hyperglycemia in critically ill patients Crit Care Med 2012 40 3251 3276 23164767 10.1097/CCM.0b013e3182653269

[B26] Qaseem A Chou R Humphrey LL Shekelle P Inpatient glycemic control: best practice advice from the clinical guidelines committee of the American college of physicians Am J Med Qual 2013 Available from: http://ajm.sagepub.com/content/early/2013/06/06/1062860613489339.abstract 10.1177/1062860613489339 23709472

[B27] Schnipper JL Barsky EE Shaykevich S Fitzmaurice G Pendergrass ML Inpatient management of diabetes and hyperglycemia among general medicine patients at a large teaching hospital J Hosp Med 2006 1 145 150 17219488 10.1002/jhm.96

[B28] Swanson CM Potter DJ Kongable GL Cook CB Update on inpatient glycemic control in hospitals in the United States Endocr Pract 2011 17 853 861 21550947 10.4158/EP11042.OR

[B29] Sociedade Brasileira de Diabetes Controle da hiperglicemia intra-hospitalar em pacientes críticos e não críticos Available from: http://www.diabetes.org.br/attachments/posicionamento/posicionamento-sbd-n-02-2011.pdf

[B30] Malerbi DA Franco LJ Multicenter study of the prevalence of diabetes mellitus and impaired glucose tolerance in the urban Brazilian population aged 30-69 yr. The Brazilian cooperative group on the study of diabetes prevalence Diabet Care 1992 15 1509 1516 10.2337/diacare.15.11.1509 1468278

[B31] Mendes AB Fittipaldi JA Neves RC Chacra AR Moreira ED Jr Prevalence and correlates of inadequate glycaemic control: results from a nationwide survey in 6,671 adults with diabetes in Brazil Acta diabetologica 2010 47 137 145 19655083 10.1007/s00592-009-0138-z PMC2859160

[B32] Fox KM Gerber Pharmd RA Bolinder B Chen J Kumar S Prevalence of inadequate glycemic control among patients with type 2 diabetes in the United Kingdom general practice research database: a series of retrospective analyses of data from 1998 through 2002 Clin Ther 2006 28 388 395 16750453 10.1016/j.clinthera.2006.03.005

[B33] Harris SB Ekoe JM Zdanowicz Y Webster-Bogaert S Glycemic control and morbidity in the Canadian primary care setting (results of the diabetes in Canada evaluation study) Diab Res Clin Pract 2005 70 90 97 10.1016/j.diabres.2005.03.024 15890428

[B34] Kobayashi M Yamazaki K Hirao K Oishi M Kanatsuka A Yamauchi M Takagi H Kawai K The status of diabetes control and antidiabetic drug therapy in Japan–a cross-sectional survey of 17,000 patients with diabetes mellitus (JDDM 1) Diab Res Clin Pract 2006 73 198 204 10.1016/j.diabres.2006.01.013 16621117

[B35] Moreira ED Jr Neves RC Nunes ZO de Almeida MC Mendes AB Fittipaldi JA Ablan F Glycemic control and its correlates in patients with diabetes in Venezuela: results from a nationwide survey Diab Res Clin Pract 2010 87 407 414 10.1016/j.diabres.2009.12.014 20060190

[B36] von Elm E Altman DG Egger M Pocock SJ GÃ¸tzsche PC Vandenbroucke JP The Strengthening the Reporting of Observational Studies in Epidemiology (STROBE) Statement: guidelines for reporting observational studies Prev Med 2007 45 247 251 17950122 10.1016/j.ypmed.2007.08.012

[B37] Goldberg PA Bozzo JE Thomas PG Mesmer MM Sakharova OV Radford MJ Inzucchi SE “Glucometrics”–assessing the quality of inpatient glucose management Diabetes Technol Ther 2006 8 560 569 17037970 10.1089/dia.2006.8.560

[B38] Queale WS Seidler AJ Brancati FL Glycemic control and sliding scale insulin use in medical inpatients with diabetes mellitus Arch Intern Med 1997 157 545 552 9066459

[B39] Wexler DJ Meigs JB Cagliero E Nathan DM Grant RW Prevalence of hyper- and hypoglycemia among inpatients with diabetes: a national survey of 44 U.S. hospitals Diabetes care 2007 30 367 369 17259511 10.2337/dc06-1715

[B40] Cobaugh DJ Maynard G Cooper L Kienle PC Vigersky R Childers D Weber R Carson SL Mabrey ME Roderman N Enhancing insulin-use safety in hospitals: practical recommendations from an ASHP Foundation expert consensus panel Am J Health Syst Pharm 2013 70 1404 1413 23903479 10.2146/ajhp130169

[B41] Cousins D Rosario C Scarpello J Insulin, hospitals and harm: a review of patient safety incidents reported to the National Patient Safety Agency Clin Med 2011 11 28 30 21404780 10.7861/clinmedicine.11-1-28 PMC5873796

[B42] Thomas AN Panchagnula U Medication-related patient safety incidents in critical care: a review of reports to the UK national patient safety agency Anaesthesia 2008 63 726 733 18582258 10.1111/j.1365-2044.2008.05485.x

