# Correction: Glycemic control and diabetes management in hospitalized patients in Brazil

**DOI:** 10.1186/1758-5996-5-78

**Published:** 2013-12-09

**Authors:** Edson Duarte Moreira, Patricia Carvalho Balthazar Silveira, Raimundo Celestino Silva Neves, Clodoaldo Souza, Zaira Onofre Nunes, Maria da Conceição C Almeida

**Affiliations:** 1 Clinical Research Center, Charitable Works Foundation of Sister Dulce, Av. Bonfim 161, Salvador, Bahia 40.415-000, Brasil; 2 Division of Cancer Epidemiology, McGill University, 546, Pine Avenue West, Montreal H2W 1S, Quebec, Canada; 3 Gonçalo Moniz Research Center, Oswaldo Cruz Foundation, Brazilian Ministry of Health, Rua Waldemar Falcão 121, Salvador, Bahia 40.296-710, Brasil

## Correction

After publication of this work [1], we noted that we inadvertently failed to include the complete list of all members who took part in the Brazilian Diabetes Investigators’ Group. The full list of members has now been added.

## Appendix

The following is a list of co-authors, the members of the Brazilian Diabetes Investigators’ Group: Adriana C. Forti, MD; Antônio R. Chacra, MD; Antônio T. F. Freire, MD; Bruno M. Camargos, MD; Célia R Nogueira, MD; Daniel Panarotto, MD; Denise D. Iezzi, MD; Edmur C. Araújo, MD; Guilherme A. F. S. Rollin, MD; Hermelinda Pedrosa, MD; João E. N. Salles, MD; João S. Felício, MD; José A. Sgarbi, MD; Luciano R. Giacaglia, MD; Marco A. Mattos, MD; Maria J. A. G. Cerqueira, MD; Miguel N. Hissa, MD; Milton M. Golbert, MD; Nubia W. Vieira, MD; Roberto A. Raduan, MD; Rubens A. Sargaço, MD; Silmara A. O. Leite, MD and Victoria Z. C. Borba.
